# Orbital mucosa-associated lymphoid tissue (MALT) lymphoma as the initial presentation in patients with hepatitis C virus infection

**DOI:** 10.5935/0004-2749.2022-0091

**Published:** 2024-02-23

**Authors:** Leyre Lloreda-Martin, Ana Berrocal-Cuadrado, Maria Angeles Torres Nieto, Alicia Galindo-Ferreiro

**Affiliations:** 1 Department of Ophthalmology, Hospital Universitario Fundación Alcorcón, Madrid, Spain; 2 Department of Ophthalmology, Rio Hortega University Hospital, Valladolid, Spain; 3 Department of Pathology, Rio Hortega University Hospital, Valladolid, Spain

**Keywords:** Orbital disease, Orbital neoplasms, Lymphoma, B-cell marginal zone, Hepacivirus, Hepatitis C, Humans, Case reports

## Abstract

Hepatitis C virus infection may be implicated in 12.7% of ocular adnexal marginal zone lymphomas. We present the first case of an orbital-systemic mucosa-associated lymphoid tissue lymphoma that responded to hepatitis C virus medical treatment. A 62-year-old male with a right-sided orbital mass was diagnosed with stage IIA orbital marginal zone lymphoma in addition to hepatitis C virus infection based on clinical, imaging, laboratory, and histological examinations. The systemic and orbital responses were achieved 1 year after undergoing hepatitis C virus treatment with glecaprevir/pibrentasvir. The association between the hepatitis C virus infection and orbital-systemic mucosa-associated lymphoid tissue lymphoma is relevant. Accordingly, patients with orbital mucosa-associated lymphoid tissue lymphoma should be assessed for hepatitis C virus seroreactivity for therapeutic and prognostic purposes.

## INTRODUCTION

Extranodal marginal zone B-cell lymphoma of mucosa-associated lymphoid tissue (MALT) is a type of low-grade non-Hodgkin’s lymphoma, which is the most common malignant orbital lesion in adults^(1)^. The appearance of MALT lymphomas is linked to environmental factors and the host’s immune response^(2)^. Moreover, a pathogenic association between some infectious agents, such as the Hepatitis C virus (HCV), and non-Hodgkin’s lymphomas (NHL), mostly MALT-type, has been reported, with HCV infection being present in 12.7% of cases of ocular adnexal marginal zone lymphomas (OAML)^(3)^.

A rare relationship between HCV and orbital lymphoma has been demonstrated^(3,4)^, but there is only one published case in which a localized orbital MALT lymphoma was resolved after HCV treatment^(4)^.

We report the first patient with a disseminated, unilateral, orbital MALT lymphoma who achieved complete systemic remission and regression of the orbital tumor after HCV treatment.

## CASE REPORT

A 62-year-old male was referred to our center with an orbital mass in his right eye (RE). The patient denied any relevant past medical or familial history, as well as any weight loss, fatigue, night sweats, or fever.

**Figure 1:**
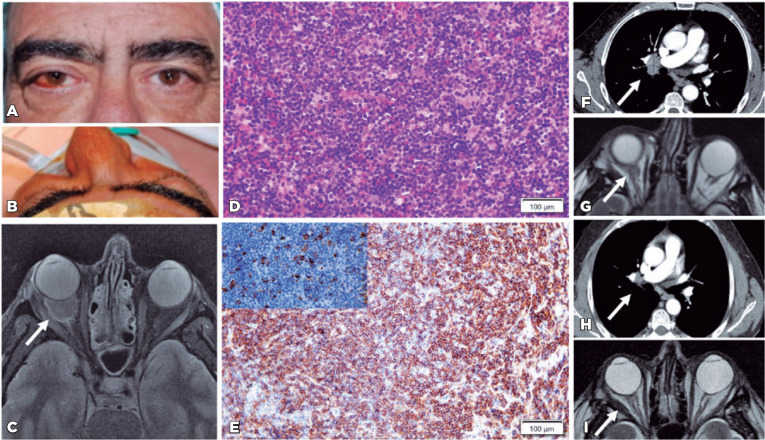
Figure composition of orbital-systemic mucosa-associated lymphoid (MALT) as the initial presentation of hepatitis C virus infection. A. Clinical appearance on the day of presentation, where he shows eyelid edema, and temporal swelling in his right eye (RE). B. Proptosis in his RE. C. Axial T2-weighted fat-suppressed magnetic resonance imaging (MRI) at presentation, showing a right intraconal mass (20 × 11 × 19 mm) surrounding the optic nerve (white arrow). D-E. Pathology slides D. Hematoxylin-eosin (HE) 40×: Diffuse infiltrate by small lymphoid cells without follicles. E. Diffuse positivity in lymphocytic B cells for CD20 (20×) and kappa light chain in some cells (left upper corner of the figure). F. Contrast-enhanced axial thoracic computed tomography (CT) at the aortic arch level, showing right paratracheal lymph node enlargement (17 mm). G. Axial T2-weighted fat-suppressed MRI, 6 months after HCV treatment, shows a decrease in the orbit tumor size (4 × 9 mm) (white arrow). H. Contrast-enhanced axial thoracic CT, 1 year after HCV treatment, shows normal paratracheal lymph nodes (arrow). I. Axial T2-weighted fat-suppressed MRI, 1 year after HCV treatment, showed the stability of the orbit tumor size, and DWI showed no cellular activity of the tumor.

The examination revealed a right upper eyelid edema and a mass on the medial canthus. A slit lamp examination revealed a red, sectoral, temporal swelling in his RE and a normal anterior segment in his left eye (LE) (Figures 1A, B). Intraocular pressure were 21 and 19 mmHg in the RE and LE, respectively. The ocular protrusion on exophtalmometry was found to be 24/21 mm. The eye motility was normal with no diplopia. Funduscopic examination was normal for both eyes.

Orbital magnetic resonance imaging (MRI) scan revealed a solid, hypercellular, and well-circumscribed intraconal mass (20 × 11 × 19 mm) surrounding and constricting the right optic nerve. The mass exhibited a homogeneous intermediate signal on T2-weighted imaging with homogeneous enhancement, a bright diffuse weight signal, and a low apparent diffusion coefficient (ADC) (Figure 1C).

Incisional biopsy and a lateral orbitotomy of the orbital mass were performed. Immunohistopathological examination of the samples showed diffuse lymphocytic infiltration of the orbital fat tissue without any follicles with germinal centers (Figure 1D). The tumor cells were positive for CD20 (Figure 1E), CD79a, PAX5, bcl2 and negative for bcl6, CD10, CD5 CD23, and cyclin D1. Some cells expressed high positivity for the light kappa chain. A MALT lymphoma with kappa-restricted positivity was diagnosed. The patient was referred for systemic investigations. The computerized tomography (CT) scans of the neck, chest, abdomen, and pelvis showed cervical, supraclavicular (60 × 50 mm), paratracheal (17 mm), and hepatic lymphadenopathy (38 mm) (Figure 1F). The bone marrow biopsy result was normal. On physical examination, there was no palpable lymphadenopathy, splenomegaly, or hepatomegaly. The laboratory tests revealed a monoclonal gammopathy of undetermined significance with monoclonal complement Ig kappa (0.13 gr/dl). The blood count and renal and liver functions were within normal limits. The patient was diagnosed with stage IIA MALT lymphoma as per the Lugano classification (2014). A polymerase chain reaction (PCR) test revealed an HCV viral load of 1.278.550 UI/mL of the 1b genotype. The patient was started on oral treatment for HCV (Glecaprevir/Pibrentasvir (Maviret^®^, Abbvie, Germany) at a dose of 100 mg/40 mg 3 times a day, for 8 weeks).

The treatment induced a viral response with undetectable HCV-RNA levels by PCR. Six months later, MRI showed a significant reduction in the orbital tumor size (4 × 9 mm) (Figure 1G), and a CT scan revealed a complete resolution of the generalized lymphadenopathy (Figure 1H). One year after starting the treatment, MRI showed a stable orbital tumor (Figure 1I).

## DISCUSSION

We present the first case of a patient with orbital--systemic MALT lymphoma and concomitant HCV infection who improved after medical treatment for HCV. A pathogenic link between HCV and lymphoma, mostly MALT, has been widely suggested, and there have been several studies reporting an increased HCV prevalence in patients with systemic NHL^(5,6)^. However, few studies have explored the association between HCV and ocular adnexal lymphomas (e.g., conjunctiva, lacrimal gland, and orbital soft tissue)^(3-5,7,8)^.

Our patient presented an orbital MALT lymphoma located around the optic nerve, with secondary eyelid edema, proptosis, and recurrent hyposphagma. Only one published report describes orbital MALT lymphoma with concurrent HCV infection with a remission of the lymphoma (localized lacrimal MALT lymphoma) after HCV medical treatment^(4)^. The peculiarity of our present case is not only that the patient had an orbital MALT lymphoma but also a disseminated disease.

When evaluating a patient with suspected orbital lymphoma, imaging (orbital and systemic), orbital biopsy^(7)^, and laboratory tests, including VHC, should be ordered.

*There is only one case reported in the literature that describes a patient with concomitant HCV and lacrimal gland MALT tumor*^(2)^ (which exhibited diffuse lymphomatous infiltration of the lacrimal gland), and high levels of liver enzymes and antibodies to HCV. However, our patient showed an intraconal mass around the optic nerve with generalized lymphadenopathy, a normal lacrimal gland, normal liver enzyme levels, and antibodies to HCV.

Various treatment modalities have been used for MALT lymphomas (chemotherapy, radiation therapy, antibiotics in the case of *Helicobacter pylori* same associated gastric MALT lymphoma, monoclonal antibody treatment, etc.)^(5)^. In our case, the patient was treated for 6 months with glecaprevir/pibrentasvir. A reduction in the size of the orbital MALT lymphoma and a complete clinical and radiological full-body remission were obtained. A good response has also been demonstrated in patients with non-orbital lymphoproliferative disorders and concomitant HCV in whom the HCV infection was treated^(4,9)^.

The new modalities for treating HCV infection have few side effects, with most of them being well tolerated. It is known, that treating the HCV infection in these patients may help diminish the size of the tumor. This fact allows us to reduce the need to use more aggressive treatments for NHL, such as chemotherapy or radiotherapy, thus improving the quality of life of our patients^(10)^. Concomitant HCV infection and MALT lymphoma are associated with more aggressive behavior and disseminated disease^(3-4)^. However, some studies of a more widespread form of the disease show that survival is not statistically different between HCV-infected and uninfected patients. After a one-year follow-up, our patient improved, and no relapse has been found. However, we still need to follow him up closely^(8)^.

In summary, the combination of orbital MALT and HCV infection is a relevant association, and to the best of our knowledge, clinical remission and radiological response after the treatment of chronic HCV in a patient with disseminated orbital MALT lymphoma have never been described before in the literature.

This case demonstrates the importance of HCV testing and treatment in all patients with orbital MALT lymphoma.
